# Draft Genome Sequence of Extended-Spectrum Beta-Lactamase-Producing *Serratia fonticola* BWK15 Isolated from Feces of *Anas penelope*

**DOI:** 10.1128/genomeA.01102-17

**Published:** 2017-10-05

**Authors:** Takehiko Kenzaka, Katsuji Tani

**Affiliations:** Environmental Science and Microbiology, Faculty of Pharmacy, Osaka Ohtani University, Nishikiori-kita, Tondabayashi, Japan

## Abstract

Migratory birds have been postulated as potential vehicles of antibiotic resistance. Here we isolated the extended-spectrum beta-lactamase (ESBL)-producing *Serratia fonticola* strain BWK15 from the feces of *Anas penelope*. The strain’s draft genome sequence indicated that it harbors class A ESBL, class C beta-lactamase, and many multidrug efflux pumps.

## GENOME ANNOUNCEMENT

Many avian species have been reported to carry drug-resistant bacteria harboring resistance genes (1). Owing to their ability to migrate long distances in short time periods, migratory birds are a potential source of antibiotic-resistant bacteria that colonize and/or infect humans (2). The Eurasian wigeon (*Anas penelope*) breeds in lowland freshwater marshes, slow-flowing rivers, and lakes in Eurasia and Northern America (3). *A. penelope* migrates from eastern Siberia to the mainland of Japan at the end of autumn and migrates back to eastern Siberia during spring (4). This migratory population is estimated to comprise hundreds of thousands of birds annually in Japan. However, the incidence and type of antibiotic-resistant bacteria that are associated with migratory birds in East Asia remain unclear.

Extended-spectrum beta-lactamase (ESBL)-producing *Serratia fonticola* BWK15 was isolated on CHROMagar ESBL medium (Kanto Chemical Co., Inc., Tokyo, Japan) from the feces of *A. penelope*, and the draft genome sequence of *S. fonticola* BWK15 was analyzed. *S. fonticola* is found in a wide range of environments, including drinking water, soil, and sewage (5, 6). *S. fonticola* has been isolated from clinical samples obtained from wounds and respiratory tracts and is regarded as a significant human pathogen causing a variety of infections (7).

Draft genomes were sequenced by 100-bp paired-end sequencing on an Illumina Hiseq 2000 sequencing system (Hokkaido System Science Co., Ltd., Sapporo, Hokkaido, Japan). High-quality sequence reads (45,069,214) were assembled *de novo* using CLC Genomics Workbench v6.5 (CLC bio, Cambridge, MA, USA). Approximately 99.6% of the sequenced reads were mapped again to the contigs. The final assembly of the genome produced 5,474,742 bp in 84 contigs with an *N*_50_ value of 184,845 bp and 53.9% GC content. The assembled contigs were functionally annotated using the RAST annotation server (8). The genomes contained 4,886 putative coding sequences (CDSs) and 72 RNA genes.

The genome of BWK15 encoded SFO family class A ESBL (CTX-M) and class C beta-lactamase. In addition, the genome encoded the following multidrug resistance proteins: streptothricin acetyltransferase, fosfomycin resistance protein, and mdtABCD multidrug resistance cluster. It also encoded the following multidrug resistance (MDR) efflux pumps: resistance-nodulation-division (RND) efflux system membrane fusion protein/inner membrane transporter/outer membrane lipoprotein (CmeA, CmeB, and CmeC), multidrug and toxic compound extrusion (MATE) family of MDR efflux pumps, multidrug efflux transporter major facilitator superfamily, macrolide-specific efflux protein MacA, macrolide export ATP-binding/permease protein MacB, and membrane fusion protein of RND family multidrug efflux pump.

These results suggest that *A. penelope* can spread multidrug-resistant *S. fonticola* through migration between Japan and eastern Siberia and that the bacteria can be transmitted from birds to humans and vice versa. The genome of *S. fonticola* BWK15 will facilitate the understanding of the ecology and global distribution of *S. fonticola* via migratory birds (9, 10). Studies on *S. fonticola* associated with *A. penelope* may help improve the understanding of antibiotic resistance dissemination in the environment.

### Accession number(s).

The draft genome sequence of the *S. fonticola* strain BWK15 has been deposited in the DDBJ/EMBL/GenBank with the accession number NQMP00000000.
